# Association between Mannose-Binding Lectin Gene Polymorphisms and Hepatitis B Virus Infection: A Meta-Analysis

**DOI:** 10.1371/journal.pone.0075371

**Published:** 2013-10-08

**Authors:** Hang-di Xu, Ming-fei Zhao, Tian-hong Wan, Guang-zhong Song, Ji-liang He, Zhi Chen

**Affiliations:** 1 Sir Run Run Shaw Hospital, School of Medicine, Zhejiang University, Hangzhou, Zhejiang, China; 2 Department of Neurosurgery, Second Affiliated Hospital, School of Medicine, Zhejiang University, Hangzhou, Zhejiang, China; 3 Institutes of Environmental Medicine, School of Medicine, Zhejiang University, Hangzhou, Zhejiang, China; 4 State Key Laboratory for Diagnosis and Treatment of Infectious Diseases, the First Affiliated Hospital, School of Medicine, Zhejiang University, Hangzhou, Zhejiang, China; MOE Key Laboratory of Environment and Health, School of Public Health, Tongji Medical College, Huazhong University of Science and Technology, China

## Abstract

**Objective:**

The results of studies on the relation between Mannose-binding lectin gene (*mbl*2) polymorphism and HBV infection were contradictory and inconclusive. In order to shed a light on these inconsistent findings and to clarify the role of *mbl*2 polymorphisms in susceptibility or progression of chronic hepatitis B (CHB), a meta-analysis was performed.

**Methods:**

PubMed and Embase were searched for available articles. A meta-analysis was performed to examine the association between *mbl*2 polymorphisms and chronicity or progression of hepatitis B infection. Odds ratio (OR) and its 95% confidence interval (CI) served as indexes.

**Results:**

A total of 17 eligible studies were involved, including 2151 healthy controls (HC), 1293 spontaneous recovered (SR) patients with acute infection, 2337cases with chronic hepatitis B (CHB) and 554 cases with progressive hepatitis B. There was no evidence of significant association between *mbl*2 exon1 polymorphisms and CHB risk in any genetic model or pairwise comparisons when compared with HC group or SR group. In the stratified analysis of ethnic groups, also no obvious relation between *mbl*2 polymorphism and CHB risk was identified. There was still no significant association between the complete *mbl*2 genotypic profile (including both the exon1 and the promoter gene) polymorphisms and CHB risk, as compared with SR group. However, it was found that there was an association between the *mbl*2 AO/OO genotype and severe hepatitis B (SHB) or liver cirrhosis (LC) (LC vs. HC:OR=3.66, 95%CI, 2.38-5.63; SHB vs. HC, OR=3.88, 95%CI, 2.26–6.64), but there was no relationship between the *mbl*2 AO/OO genotype and hepatocellular carcinoma (HCC) (OR=1.26, 95%CI, 0.82-1.94).

**Conclusion:**

The present meta-analysis indicated that *mbl*2 exon1 polymorphisms might not significantly associate with chronicity of HBV infection, but might be significantly related to the progressive HBV such as SHB and LC.

## Introduction

Hepatitis B virus (HBV) infection leads to a wide spectrum of clinical presentations from inapparent infection to self-limiting acute hepatitis, chronic infection, fulminant hepatic failure (FHF), liver cirrhosis (LC) and hepatocellular carcinoma (HCC) [1]. The mechanism for persistent and progressive HBV infection is still unclear, but host immune factors and genetic factors may play important roles [2].

Mannose-binding lectin (MBL) is an important constituent of the human innate immune system, which, as an acute-phase reactant, is secreted by the liver. MBL is a calcium-dependent C-type lectin with a structural analogy of complement component C1q. MBL can bind through multiple lectin domains to the carbohydrate moieties expressed on the surface of many microbial organisms, and activate macrophages and the complement system cascade [3]. It is also reported that serum MBL play an important role in regulating the production of proinflammatory cytokines such as TNF-α, IL-6 and IL-1βby monocytes in response to microbial infection [4], hence may affect the inflammation severity or disease progression.

Polymorphisms in the MBL gene (*mbl*2) have been shown to affect the oligomer formation and circulating levels of MBL [5]. Three single nucleotide polymorphisms (SNPs) in exon1 (codon52, codon54, and codon57) of *mbl*2 gene give rise to amino acid substitutions within the collagen-like region of MBL. These three polymorphic alleles are collectively designated as allele O, while the wild-type structural allele is described as allele A [6]. The wild-type A/A, heterogeneous-type A/O and homologous-type O/O are generally associated with high, intermediate and low (or absent) MBL levels, respectively. SNPs have also been found in the promoter region of *mbl*2 at positions 221 Y/X [7], and the X variant also significantly reduces the serum MBL levels. In recent years, a number of clinical and genetic studies demonstrated the association between low serum MBL levels due to SNPs in *mbl*2 and CHB [8-24]. Some studies reported that patients with *mbl*2 mutations are prone to developing persistent HBV infection and that the MBL plays an important role in the clinical outcome after HBV infection [8,11,18]. Moreover, it was reported that the *mbl*2 polymorphisms may be an important factor in determining the prognosis in patients with hepatitis B virus infection [13]. However, the data from several studies argue against a role of MBL in chronicity and progression of HBV [9,17]. For reason given above, a meta-analysis in a large population was fulfilled in present study.

## Materials and Methods

### Search strategy

In order to collect all papers involved in *mbl*2 polymorphisms and HBV, PubMed and Embase (up date to 31 March 2013) were searched independently by two investigators (Hangdi Xu and Mingfei Zhao). The search terms were the following key words: (“Mannose binding lectin” or “Mannose binding protein” or “Mannose-binding lectin” or “Mannose-binding protein” or “MBL2” or “MBP2” or “MBL-2” or “MBP-2”), and (“HBV” or “Hepatitis B”). The search strategy was shown in Table 1. The reference lists of reviews and retrieved articles were searched at the same time by hand. Data were only recruited from the full published paper, and did not include any conference abstract or unpublished report. When the same patient population was included in several publications, only the one with the biggest size sample or one in the most recent study was used in present meta-analysis.

**Table 1:** Search strategy for electronic databases.

**PubMed**
("mannose-binding lectin"[MeSH Terms] OR "mannose-binding lectin"[All Fields] OR "mannose binding lectin"[All Fields] OR "mannose-binding protein"[All Fields] OR "mannose binding protein"[All Fields] OR "mbl2"[All Fields] OR "mbl-2"[All Fields] OR "mbp-2"[All Fields] OR "mbp2"[All Fields]) AND (hbv[All Fields] OR "hepatitis b"[MeSH Terms] OR "hepatitis b"[All Fields])
**Embase**
('mannose-binding lectin'/exp OR 'mannose-binding lectin' OR 'mannose binding lectin'/exp OR 'mannose binding lectin' OR 'mannose-binding protein'/exp OR 'mannose-binding protein' OR 'mannose binding protein'/exp OR 'mannose binding protein' OR 'mbl2' OR 'mbl-2' OR 'mbp-2' OR 'mbp2') AND ('hbv’/exp OR hbv OR 'hepatitis b'/exp OR 'hepatitis b')

### Criteria of inclusion and exclusion

The criteria of collecting the published studies were following: (1) the evaluation of the association between *mbl*2 polymorphisms and HBV infection; (2) a case-control or cohort studies; (3) sufficient genotype data for calculating the odds ratio (OR) with 95% confidence interval (CI). The criteria of excluding studies were following: (1) the studies with overlapped data; (2) abstracts, comments, reviews or editorial letters.

### Data extraction

The following information was extracted from each study: the first author’s name, years of publication, ethnicity of population, numbers of cases and controls, genotyping methods, genotyping results of cases and controls, and the Hardy-Weinberg equilibrium (HWE) results. The control groups included healthy control (HC) groups and spontaneous recovered (SR) groups with acute infection.

### Definitions

HC were negative for hepatitis B surface antigen (HBsAg) and HBV DNA. The spontaneously recovered (SR) individuals were positive for both hepatitis B surface antibody and hepatitis B core antibody, but definitely negative for HBsAg; or positive for HBsAg and IgM anti-HBc on presentation and clearing HBsAg within 6 months. CHB individuals were with persistent HBsAg positive for more than 6 months. SHB patients had severe liver disease symptoms, including obvious clinical manifestations and conspicuous changes in their biochemical parameters, such as significant serum alanine aminotransferase (ALT) elevation (more than 5 folds of normal levels) and plasma prothrombin activity (PTA) <60%. LC subjects were defined as HBsAg carriers whose liver ultrasound scan or liver function test confirmed cirrhosis. HCC patients were diagnosed by serum α-fetoprotein levels and hepatic arteriography or histology.

### Statistical analysis

First of all, HWE was assessed in the HC groups using the goodness-of-fit test (χ^2^ or Fisher’s exact test) and a *P* < 0.05 was considered as significant disequilibrium. OR and the corresponding 95% CI were utilized to evaluate the strength of association of *mbl*2 polymorphisms with CHB. Quantitative meta-analysis was performed using STATA version 10.0 (STATA Corporation, College Station, TX, USA). Heterogeneity was assessed for each study using Cochrane’s Q-test and *I* ^2^ measurement (*I* ^2^ is defined as the proportion of total variations across studies, which are due to heterogeneity rather than chance). *P* ≤ 0.10 or I^2^ ≥ 50% indicated that the heterogeneity was significant. A random effect model was used in the presence of substantial heterogeneity. The potential risk of publication bias was examined by the Egger test, and *P* ≤ 0.10 indicates the presence of a publication bias. Subgroup analysis was also performed for ethnicity and the types of progressive hepatitis B virus infection. Sensitivity analysis was conducted to evaluate the validity and reliability of primary meta-analysis. Nonsuperiority test was used to confirm the absence of association between mbl2 exon 1 polymorphism and persistence of HBV infection.

## Results

### Characteristics of studies included in the meta-analysis

**Figure 1:**
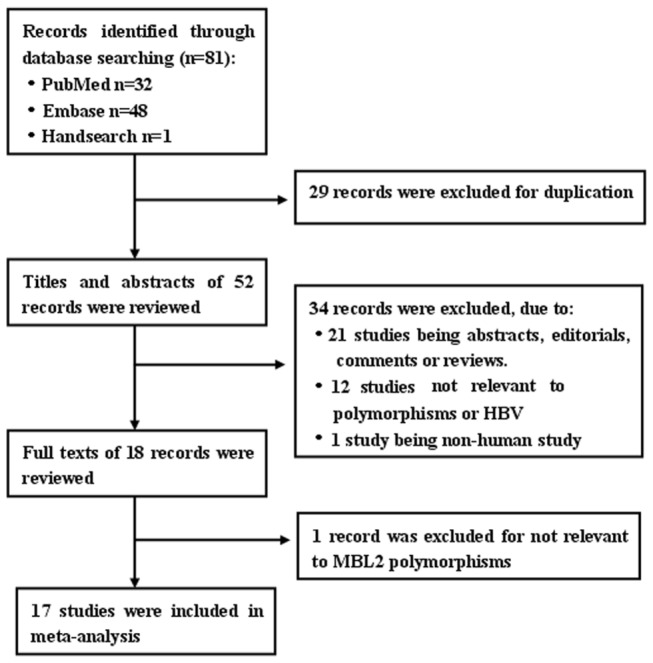
Flow chart for selection of studies.

After searching the databases, 81 potential eligible reports were identified (Figure 1), and 29 records were excluded due to duplicate data, and 21 records, which belonged to abstracts, editorials, comments or reviews, were excluded. Also 13 studies not related to polymorphisms or HBV were excluded. One record, a non-case control study, was excluded. Finally, a total of 17 studies were involved, which included 2151 HC, 1293 SR patients with acute infection, 2337cases with CHB and 554 cases (175 cases with severe hepatitis B (SHB), 223 cases with LC and 156 cases with HCC). Table 2 showed the general characteristics of the included studies.

**Table 2:** Characteristics of studies for association between *mbl*2 polymorphisms and HBV infection.

**Study**	**Ethnicity**	**Control**	**Cases**	**Polymorphisms included**	**Genotyping Methods**	**HWE**
Thomas HC 1996 [8]	Caucasian	HC/SR	CHB	Condon52, 54 and57	SSOP; sequencing	No
	Asian	HC	CHB	Condon52, 54 and57	SSOP; sequencing	Yes
Bellamy R 1998 [9]	African	HC/SR	CHB	Condon52, 54 and57	SSOP	Yes
Höhler T 1998 [10]	Caucasian	HC/SR	CHB	Condon52 and 54	PCR-RFLP	Yes
Yuen MF 1999 [11]	Asian	HC	CHB/LC/HCC	Condon54	PCR-RFLP	−
Shi H 2001 [12]	Asian	HC	CHB	Condon52, 54 and 57	PCR-RFLP	Yes
Hakozaki Y 2002 [13]	Asian	HC	SHB	Condon52, 54 and 57; -221Y/X	sequencing	No
Song le H 2003[1]	Asian	HC	CHB/LC/HCC	Condon52, 54 and57	sequencing	Yes
Cheong JY 2005 [14]	Asian	SR	CHB	Condon54	SBE	Yes
Thio CL 2005[15]	A mixed population	SR	CHB	*Mbl2* promoter and exon1 combined polymorphisms	Real time PCR	−
Chong WP 2005 [16]	Asian	HC/SR	CHB/LC/HCC	*Mbl*2 promoter and exon1 combined polymorphisms	TaqMan PCR	Yes
Segat L 2008 [17]	Caucasian	HC	HCC	Condon52, 54 and57	Real time PCR	Yes
Tong FY 2008 [18]	Asian	HC	CHB/SHB	Condon52, 54 and 57	sequencing	−
Filho RM 2010 [19]	A mixed population	HC	CHB	Condon52, 54 and 57; -221Y/X	Taqman PCR	Yes
Fletcher GJ 2010 [20]	A mixed population	SR	CHB	Condon52, 54 and 57; -221Y/X	PCR-SSP	−
Chen DQ 2010 [21]	Asian	SR	CHB	Condon54 and -221Y/X	PCR-RFLP	Yes
Chatzidaki V 2012 [22]	Caucasian	HC/SR	CHB	Condon54 and 57; -221Y/X	PCR-RFLP	Yes
Zheng RD 2012 [23]	Asian	HC/SR	CHB/SHB/LC	Condon54	PCR-RFLP	Yes

RFLP: restriction fragment length polymorphisms; SSOP: *sequence specific oligonucleotide* probes; PCR-SSP: polymerase chain reaction-sequence specific primer; SBE: *Single Base Primer Extension Assay*. HWE: Hardy-Weinberg equilibrium; − not available. HC: healthy control; SR: spontaneous recovered control; CHB: chronic hepatitis B; LC: liver cirrhosis; HCC: hepatocellular carcinoma; SHB: severe hepatitis B.

### Association between HBV persistence and MBL exon1 polymorphisms

**Figure 2:**
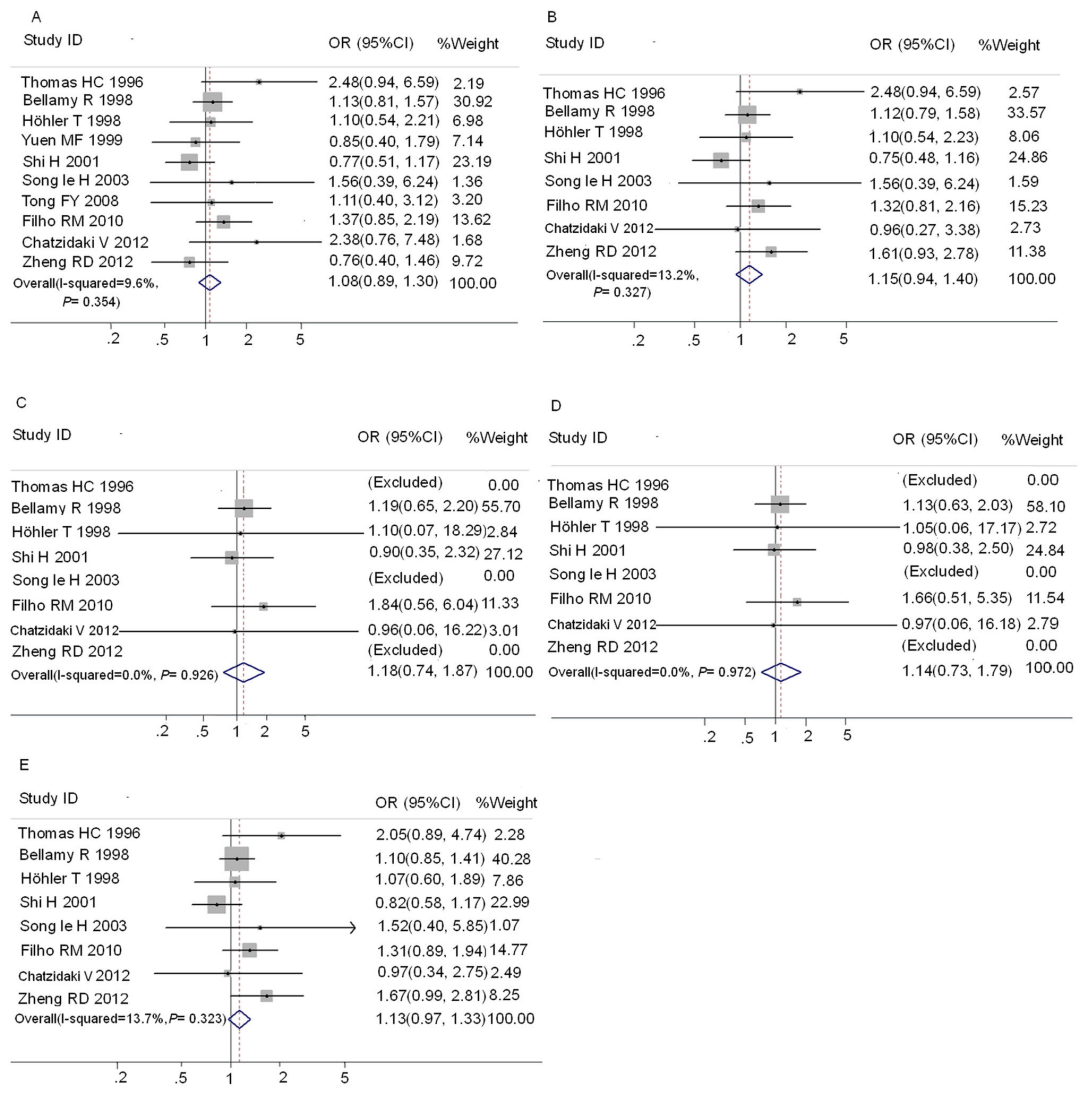
Forest plot of chronic hepatitis B virus infection risk associated with *mbl*2 exon1 polymorphisms as compared with HC. A: AO/OO vs. AA; B: AO vs. AA; C:OO vs. AA; D:OO vs. AO/AA; E: O allele vs. A allele.

**Figure 3:**
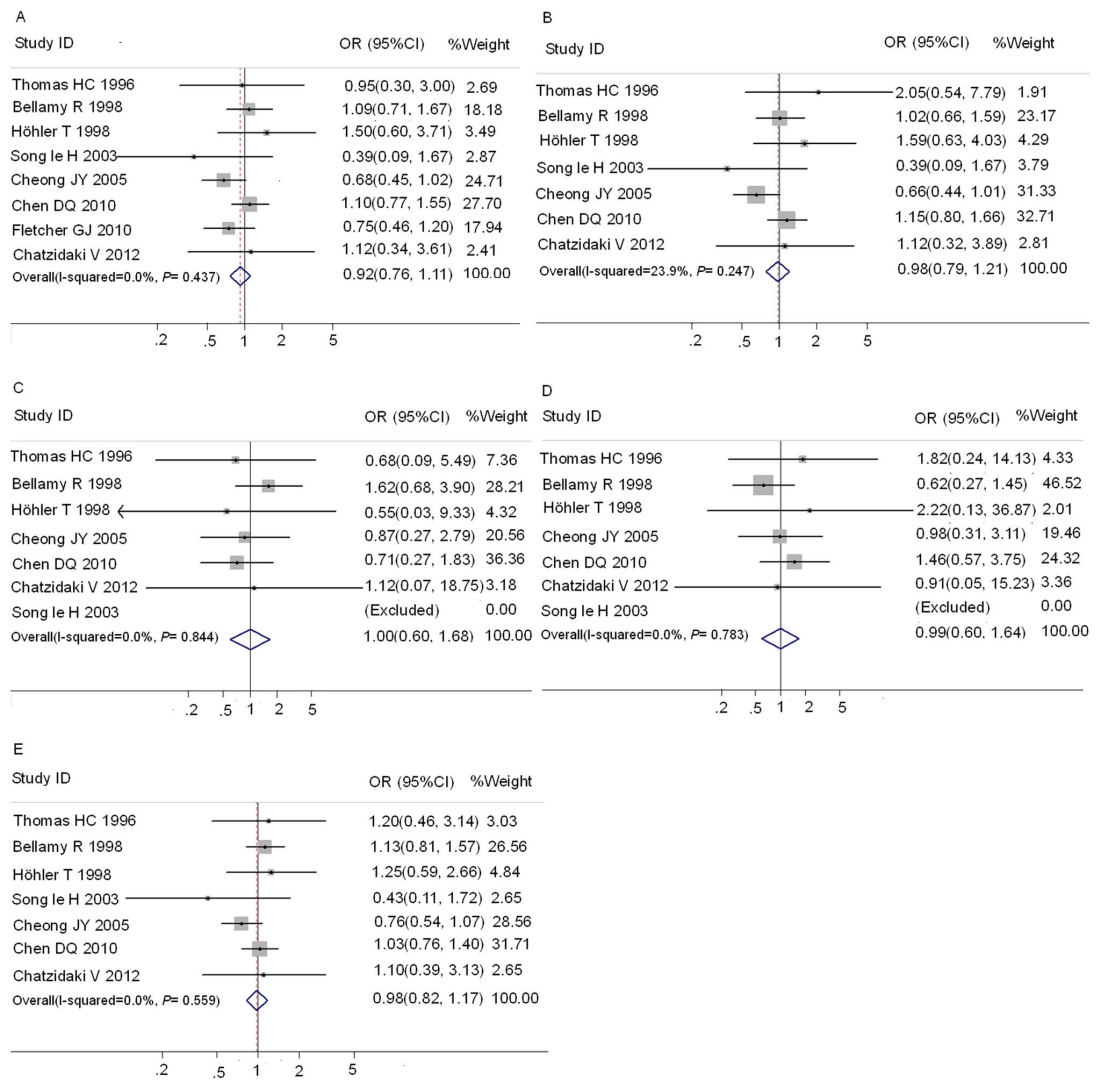
Forest plot of chronic hepatitis B virus infection risk associated with *mbl*2 exon1 polymorphisms as compared with SR group. A: AO/OO vs. AA; B: AO vs. AA; C:OO vs. AA; D:OO vs. AO/AA; E: O allele vs. A allele.

Ten studies [1,8-12,18,19,22,23] contained sufficient data for analysis of wild-type (AA) versus any MBL2 variant allele (OA/OO) genotype between HC groups and CHB groups (Table S1). The distribution of genotypes in HC of Caucasian subjects from the study of Thomas HC et al [8] was not consistent with HWE, so only the Asian subjects from this study were included in the meta-analysis between HC and CHB. Eight studies [1,8-10,14,20-22] contain sufficient data for analysis of AA versus OA/OO between SR groups and CHB groups (Table S1). Results of pooled analysis on the associations between MBL exon1 polymorphisms and the risk of CHB were shown in Figure 2 and Figure 3. Overall, no significant association between exon1 gene polymorphisms and the risk of CHB was observed as compared with both HC (Figure 2) and SR groups (Figure 3). There was no significant heterogeneity in these analyses (I^2^≤23.9%). The publication bias of these studies was assessed by Egger’s test. In HC groups, the funnel plot showed the asymmetry in *mbl*2 exon1 OO vs. AA and AO+OO vs. AA models to some extent, and Egger’s test proved the existence of publication bias in these two models (*P*= 0.020 and 0.007, respectively). No significant publication bias was detected in other comparisons.

The results of subgroup analysis based on ethnicity indicated that *mbl*2 O allele carriers (AO and / or OO) in both Asian populations and non-Asian populations were not associated with the increased risk of chronic HBV infection as compared with HC or SR groups, respectively (Table 3).

**Table 3:** Association between *mbl*2 exon1 polymorphisms and the persistence of HBV infection in different ethnicity-subgroups.

	**No. studies**	OR* for variant genotype (95% CI)		**Test for heterogeneity *P* value**	**I^2^**
**Healthy controls**					
***Asian***					
AO/OO vs AA	6	1.08	0.82-1.42	0.156	37.6%
AO vs AA	4	1.13	0.83-1.55	0.054	60.7%
OO vs AA	4	0.90	0.35-2.32	−	−
OO vs AO/AA	4	0.98	0.38-2.50	−	−
***Non-Asian***					
AO/OO vs AA	5	1.18	0.92-1.51	0.899	0.0%
AO vs AA	4	1.16	0.90-1.50	0.934	0.0%
OO vs AA	4	1.28	0.76-2.16	0.927	0.0%
OO vs AO/AA	4	1.20	0.72-1.99	0.947	0.0%
**Spontaneous recovered controls**					
***Asian***					
AO/OO vs AA	3	1.15	0.88-1.49	0.120	52.9%
AO vs AA	3	0.89	0.68-1.16	0.080	60.5%
OO vs AA	3	0.77	0.37-1.59	0.788	0.0%
OO vs AO/AA	3	0.80	0.39-1.64	0.604	0.0%
***Non-Asian***					
AO/OO vs AA	5	1.00	0.75-1.32	0.561	0.0%
AO vs AA	4	1.17	0.81-1.68	0.691	0.0%
OO vs AA	4	1.32	0.63-2.76	0.807	0.0%
OO vs AO/AA	4	1.27	0.62-2.60	0.688	0.0%

OR: odds ratio; CI: confidence interval; −: can’t be calculated. “AO/OO vs. AA”: “Dominant model”; “OO vs. AO/AA”: “Recessive model”; “AO vs. AA” and “OO vs. AA”: “Co-dominant model.

### Association between *mbl*2 promoter -221 Y/X polymorphisms and CHB

Four studies with SR groups and two studies with HC groups about association between *mbl*2 promoter -221 Y/X polymorphisms and CHB were reported (Table S2). The results of pooled analysis demonstrated no significant association between *mbl*2 promoter polymorphisms and the risk of CHB as compared with SR group (XY/XX vs. YY:OR=1.10, 95%CI 0.90-1.35; XY vs. YY:OR=0.93, 95%CI 0.71-1.21; XX vs. YY:OR=0.81, 95%CI 0.43-1.54; XX vs. XY/Y: OR=0.80, 95%CI 0.42-1.51) (Table 4). When analyzing the association between promoter polymorphisms and the risks of CHB as compared with the HC group, it was found that there was the significant relation between -221 Y/X polymorphisms and the risk of CHB under XY vs. YY contrast (OR=1.61, 95%CI 1.01-2.57), but no significant relation under other contrasts (XY/YY vs. YY:OR=1.42, 95%CI 0.91-2.20; XX vs YY:OR=0.65, 95%CI 0.23-1.90; XX vs. XY/Y: OR=0.56, 95%CI 0.20-1.61) was observed (Table 4).

**Table 4:** Meta-analysis for the association between *mbl*2 promoter -221Y/X polymorphisms and the persistence of HBV infection.

	**No. of studies**	OR* for variant genotype (95% CI)		**Test for heterogeneity *P* value**	**I^2^**
**Healthy controls**					
XY/XX vs YY	2	1.42	0.91-2.20	0.775	0.0%
XY vs YY	2	1.61	1.01-2.57	0.743	0.0%
XX vs YY	2	0.65	0.23-1.90	0.524	0.0%
XX vs XY/YY	2	0.56	0.20-1.61	0.488	0.0%
**Spontaneous recovered controls**					
XY/XX vs YY	4	1.10	0.90-1.35	0.193	36.6%
XY vs YY	3	0.93	0.71-1.21	0.640	0.0%
XX vs YY	3	0.81	0.43-1.54	0.517	0.0%
XX vs XY/YY	3	0.80	0.42-1.51	0.573	0.0%

OR: odds ratio; CI: confidence interval. “XY/XX vs. YY”: “Dominant model”; “XX vs. XY/YY”: “Recessive model”; “XY vs. YY” and “XX vs. YY”: “Co-dominant model.

### Association between *mbl*2 promoter and exon1 combined polymorphisms and CHB

**Figure 4:**
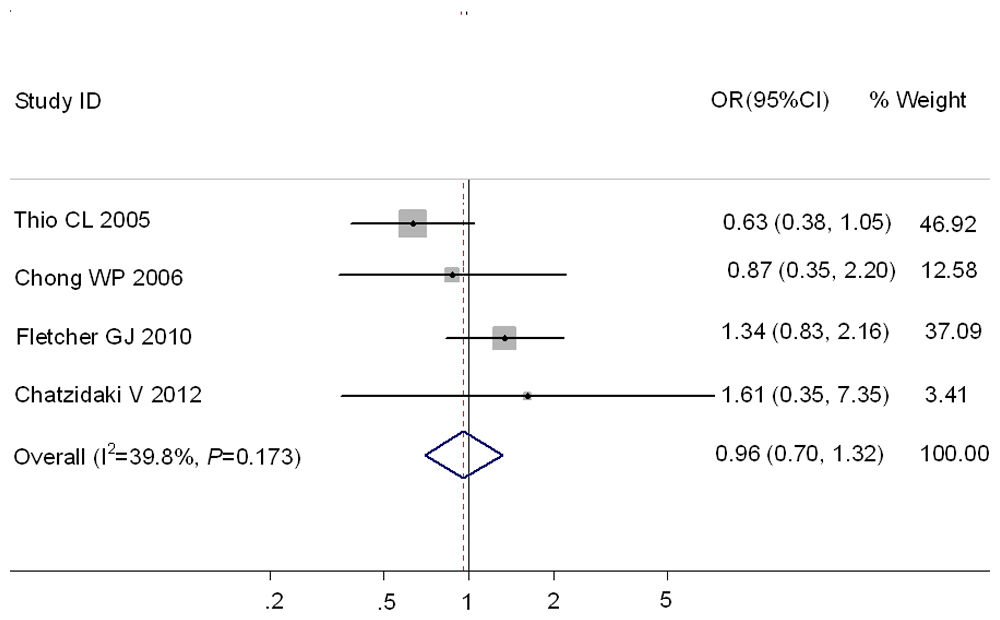
Forest plot of chronic hepatitis B virus infection risk associated with *mbl*2 promoter and exon1 combined polymorphisms as compared with SR group.

Subsequently, a meta-analysis was performed on the studies that reported a complete *mbl*2 genotypic profile including both the exon1 and the promoter polymorphisms. Although only four studies reported such data (Table S3), it was proposed that such genotype profiles were associated much more strongly with serum MBL levels than exon1 genotypes or promoter genotypes alone [24,25]. In this subset, the individuals with O/O and XA/O were considered as the subjects with low MBL production, whereas the individuals with other genotypes were demonstrated as the subjects with high MBL production [26]. However, it was found in Figure 4 that there was no significant association between *mbl*2 genotypes and the susceptibility of CHB, as compared with SR subjects (OR=0.96, 95%CI 0.70-1.32).

### Association between *mbl*2 exon1 polymorphisms and the progression of CHB

**Figure 5:**
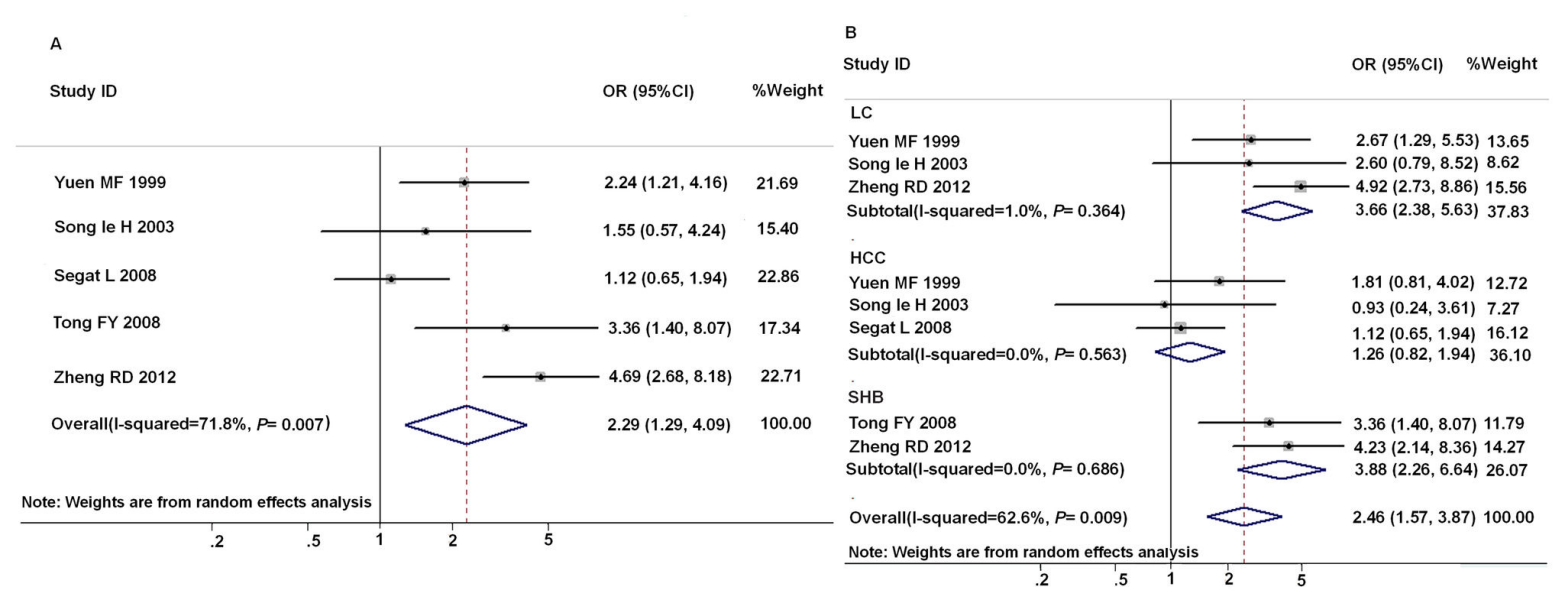
Forest plot of progressive hepatitis B disease risk associated with *mbl*2 exon1 polymorphisms as compared with HC. A: association between *mbl*2 genotypes AO/OO and HBV progressive liver diseases; B: subgroup analysis based on types of diseases.

There were six studies on hepatitis B progression with the sufficient data of *mbl*2 wild type versus *mbl*2 variant (AO/OO) genotype. As the HC group in the study of Hakozaki Y et al [13] was not consisting with HWE, so it was excluded from the meta-analysis. At last, a total of 511 cases and 545 controls were included in this analysis. As shown in Figure 5A, *mbl*2 genotypes AO/OO were significantly related to HBV progressive liver diseases (OR=2.29; 95%CI 1.29-4.09). As the progressive liver disease contains three types of diseases (SHB, LC and HCC), the association between *mbl*2 exon1 polymorphisms and the risks of each hepatic disease was investigated. The results (Figure 5B) demonstrated that there was the significant relation between *mbl*2 AO/OO genotypes and LC and SHB risks (LC vs. HC:OR=3.66, 95%CI 2.38-5.63; SHB vs. HC, OR=3.88, 95%CI, 2.26–6.64). However, the significant association between *mbl*2 AO/OO genotypes and HCC was not observed (OR=1.26, 95%CI 0.82-1.94).

### Equivalence-based analysis to confirm the lack of relationship between MBL2 polymorphism and persistence of HBV infection

Non-superiority test was performed to confirm the absence of association between MBL2 polymorphism and persistence of HBV infection. The null hypothesis is that the frequency of MBL2 exon1 O alleles in CHB patients is greater by Δ as compared with the frequency in controls. The specified amount Δ may be arbitrary, and it may be taken from the lower boundary of the difference estimated in the initial report of the genetic association [27,28]. The difference of MBL2 O allele between CHB and HC patients first reported by Thomas HC were 10.1% [8]. Thus, a 5% excess in cases represents a fairly stringent cut-off.

The corresponding nonsuperiority *P*-values for MBL2 exon1 was 0.0005 and 0.0012, as compared with HC or SR respectively, which support the absence of association between MBL2 exon1 polymorphism and persistence of HBV (Table S4 and Table S5).

## Discussion

MBL is an innate immune system pattern recognition protein which can kill the different pathogenic microbes through complement activation. The *mbl*2 polymorphisms could result in the MBL deficiency among a certain percentage of human, which potentially increases susceptibility to infectious disease [29,30]. Our meta-analysis has demonstrated no significant relation between *mbl*2 genotype and CHB. In Asia, the infection usually occurs at birth, and the infecting inoculums may be larger. About 90% of newborns, whose mothers are positive for HBeAg, will become persistently infected as a result of the induction of T-cell tolerance by secreted HBe, which crosses the placenta [31]. Thus, the genetic factors will be less important in these patients. However, among Caucasian and African patients, the infection usually occurs in adulthood and childhood, and 5% and 20% of cases become persistently infected, respectively [8]. The results of subgroup analysis based on ethnicity were shown in table 3. There was still no significant association between the chronicity of hepatitis B and *mbl*2 polymorphisms in Asian and non-Asian, respectively. However, there was the significant relation between -221 X/Y polymorphisms and the risk of CHB, when compared with HC under XY vs. YY contrast. As the studies included was limited (only two), more studies should been taken to verified this result in the future.

In order to investigate these alternative results, the additional ad hoc meta-analysis was performed for the studies that reported complete *mbl*2 genotypic profile, including promoter region and structural region. No significant association between complete *mbl*2 genotype and CHB susceptibility was found, as compared with SR group.

Our meta-analysis demonstrated that there was the significant relation between *mbl*2 variant genotypes (AO/OO) and SHB or LC. It was concluded that MBL would not appear to be involved significantly in host susceptibility to CHB, but might play an important role in exacerbation and progression of hepatitis B. These genotypes (AO/OO) might be associated with the natural wound healing process, which may develop the necroinflammation, rather than carcinogenesis [16,17].

The progression of HBV infections resulted from MBL mutations may be explained by two possible mechanisms. Firstly, MBL may have a direct effect on HBV infection through complement activation. The amannose-rich oligosaccharide was detected in the preS2 region of the hepatitis B surface protein, so MBL could bind HBV in theory [16,32]. Moreover, Chong WP et al [16] have studied the binding of MBL to HBsAg and found that MBL could bind HBsAg via its multiple carbohydrate recognition domains. It was suggested that MBL might function as an opsonin for HBV. Also MBL plays an important role in the complement system by acting as the recognition molecule of the lectin pathway and that MBL activates complement on HBsAg-MBL complexes through the lectin complement pathway and therefore may be involved in HBV clearance [16]. Besides the possibility of direct clearance of HBV, MBL-mediated complement activation might also be involved in immune complex removal during HBV infection. It was reported that immune complexes levels increased in chronic liver disease, which might be associated with the increased inflammatory responses in liver damage [33]. Thus, low MBL levels secondary to mutations may lead to defective complement activation, and poor clearance of immune complexes with subsequent deposition in the liver, which may subsequently predispose the patients to greater immunological damage of the liver [11]. The second possible mechanism is that MBL may regulate the production of some inflammatory cytokines. The high concentrations of MBL profoundly decreased the interleukin (IL)-6, IL-1β, and tumor necrosis factor-α produced by monocytes in response to pathogen, whereas lower concentrations of MBL could enhance the production of IL-6 and IL-1β [34]. So MBL not only is involved in complement activation but also is a potent regulator of inflammatory pathways, as such, MBL may affect the severity of HBV. These two mechanisms would explain why patients with *mbl*2 AO/OO genotypes were more likely to develop the symptomatic cirrhosis and severe hepatitis.

In spite of providing the most comprehensive assessment of the association between the outcome of HBV infection and the *mbl*2 polymorphisms, there are still some limitations that should be taken in to account when interpreting our results. Heterogeneity and confounding factors may have affected the meta-analysis. The moderate and high heterogeneity existed in the analyses, especially in the subgroup of Asians or the SHB group. Publication bias may be other influence factor of this meta-analysis. It is possible that the studies with negative results have not been published, and Egger’s test indicated that the publication bias existed in two contrasts when CHB group was compared with the HC group. Furthermore, there was only a small amount of data in present meta-analysis, especially in some subgroup analysis.

In summary, the association between the *mbl*2 polymorphisms and the HBV infection was systematically analyzed in present meta-analysis. The combined results showed that *mbl*2 exon1 polymorphisms might be not significantly associated with chronicity of HBV infection, but might be significantly associated with the progressive HBV such as SHB and LC. Moreover, *mbl*2 polymorphisms might be not significantly associated with carcinogenesis of CHB. Since the potential confounders could not be ruled out completely and the studies in subgroup analysis were limited, it is necessary to confirm these results with more studies in future.

## Supporting Information

Table S1 **Distribution of MBL2 exon1 genotypes among HBV cases and controls in the meta-analysis.**
(DOC)

Table S2 **Distribution of MBL2 promoter genotypes among HBV cases and controls in the meta-analysis.**
(DOC)

Table S3 **Distribution of polymorphisms of MBL2 promoter plus exon1 among HBV cases and controls in the meta-analysis.**
(DOC)

Table S4 **Non-superiority tests of mbl2 polymorphism in CHB as compared with HC.**
(DOC)

Table S5 **Non-superiority tests of mbl2 polymorphism in CHB as compared with SR.**
(DOC)

Checklist S1 **PRISMA 2009 Checklist.**
(DOC)
