# Supplementary material for: Association between Mannose-Binding Lectin Gene Polymorphisms and Hepatitis B Virus Infection: A Meta-Analysis
Source: PLoS One. 2013 Oct 8;8(10):e75371. doi: 10.1371/journal.pone.0075371 (PMC3792921; doi:10.1371/journal.pone.0075371)
Supplement: Table S1 — Distribution of MBL2 exon1 genotypes among HBV cases and controls in the meta-analysis. (DOC) [file pone.0075371.s001.doc]

Table S1 Distribution of MBL2 exon1 genotypes among HBV cases and controls in the meta-analysis.

| **Study** | **Samples** | **Total Number** | **Genotypes** | | | |
| --- | --- | --- | --- | --- | --- | --- |
|  |  |  | **OO** | **AO** | **AA** | **OO+AO** |
| **Thomas HC 1996^[8]^** |  |  |  |  |  |  |
| Caucasian | HC | 98 | 0 | 37 | 61 | 37 |
|  | SR | 19 | 2 | 4 | 13 | 6 |
|  | CHB | 33 | 2 | 12 | 19 | 14 |
| Asian | HC | 117 | 0 | 29 | 88 | 29 |
|  | CHB | 20 | 0 | 9 | 11 | 9 |
| **Bellamy R 1998^[9]^** | HC | 653 | 52 | 291 | 310 | 343 |
|  | SR | 157 | 9 | 75 | 73 | 84 |
|  | CHB | 180 | 16 | 84 | 80 | 100 |
| **Höhler T 1998^[10]^** | HC | 64 | 1 | 29 | 34 | 30 |
|  | SR | 28 | 1 | 10 | 17 | 11 |
|  | CHB | 61 | 1 | 29 | 31 | 30 |
| **Yuen MF 1999^[11]^** | HC | 117 | -- | -- | 90 | 27 |
|  | CHB | 64 | -- | -- | 51 | 13 |
|  | LC | 45 | -- | -- | 25 | 20 |
|  | HCC | 37 | -- | -- | 24 | 13 |
| **Shi H 2001^[12]^** | HC | 150 | 7 | 47 | 96 | 54 |
|  | CHB | 285 | 13 | 73 | 199 | 86 |
| **Hakozaki Y 2002^[13]^** | HC | 260 | 16 | 50 | 194 | 66 |
|  | SHB | 43 | 4 | 7 | 32 | 11 |
| **Song le H 2003^[1]^** | HC | 112 | 0 | 9 | 103 | 9 |
|  | SR | 31 | 0 | 8 | 23 | 8 |
|  | CHB | 25 | 0 | 3 | 22 | 3 |
|  | LC | 27 | 0 | 5 | 22 | 5 |
|  | HC | 40 | 0 | 3 | 37 | 3 |
| **Cheong JY 2005^[14]^** | SR | 126 | 4 | 52 | 70 | 56 |
|  | CHB | 372 | 12 | 119 | 241 | 131 |
| **Segat L 2008^[17]^** | HC | 164 | 13 | 49 | 102 | 62 |
|  | HCC | 79 | 2 | 30 | 47 | 32 |
| **Tong FY 2008^[18]^** | HC | 64 | -- | -- | 55 | 9 |
|  | CHB | 52 | -- | -- | 44 | 8 |
|  | SHB | 62 | -- | -- | 40 | 22 |
| **Filho RM 2010^[19]^** | HC | 232 | 7 | 78 | 147 | 85 |
|  | CHB | 102 | 5 | 40 | 57 | 45 |
| **Fletcher GJ 2010^[20]^** | SR | 147 | -- | -- | 79 | 68 |
|  | CHB | 133 | -- | -- | 81 | 52 |
| **Chen DQ 2010^[21]^** | SR | 361 | 12 | 79 | 270 | 91 |
|  | CHB | 304 | 7 | 75 | 222 | 82 |
| **Chatzidaki V 2012^[22]^** | HC | 33 | 1 | 6 | 25 | 7 |
|  | SR | 36 | 1 | 6 | 29 | 7 |
|  | CHB | 33 | 1 | 6 | 26 | 7 |
| **Zheng RD 2012^[23]^** | HC | 88 | 0 | 26 | 62 | 26 |
|  | CHB | 174 | 0 | 70 | 104 | 70 |
|  | LC | 151 | 1 | 95 | 55 | 96 |
|  | SHB | 70 | 1 | 41 | 28 | 42 |

HC: healthy control; SR: spontaneous recovered control; CHB: chronic hepatitis B; LC: liver cirrhosis; HCC: hepatocellular carcinoma; SHB: severe hepatitis B.
