# Supplementary material for: Association between Mannose-Binding Lectin Gene Polymorphisms and Hepatitis B Virus Infection: A Meta-Analysis
Source: PLoS One. 2013 Oct 8;8(10):e75371. doi: 10.1371/journal.pone.0075371 (PMC3792921; doi:10.1371/journal.pone.0075371)
Supplement: Table S2 — Distribution of MBL2 promoter genotypes among HBV cases and controls in the meta-analysis. (DOC) [file pone.0075371.s002.doc]

Table S2 Distribution of MBL2 promoter genotypes among HBV cases and controls in the meta-analysis.

| **Study** | **Samples** | **Total Number** | **Genotype** | | | |
| --- | --- | --- | --- | --- | --- | --- |
|  |  |  | **XX** | **XY** | **YY** | **XX+XY** |
| **Filho RM 2010^[19]^** | HC | 232 | 9 | 58 | 165 | 67 |
|  | CHB | 102 | 3 | 35 | 64 | 38 |
| **Chen DQ 2010^[21]^** | SR | 361 | 10 | 100 | 251 | 110 |
|  | CHB | 304 | 9 | 95 | 200 | 104 |
| **Fletcher GJ 2010^[20]^** | SR | 137 | 12 | 57 | 68 | 69 |
|  | CHB | 133 | 6 | 51 | 76 | 57 |
| **Chatzidaki V 2012^[22]^** | HC | 32 | 5 | 6 | 21 | 11 |
|  | SR | 36 | 2 | 13 | 21 | 15 |
|  | CHB | 33 | 2 | 11 | 20 | 13 |
| **Thio CL 2005^[15]^** | **Samples** | **Genotype XX+XY (%)** | | **OR(95%CI)** | | ***P*** |
|  | SR | 17.7 | | 1.38 (1.01–1.89) | | 0.04 |
|  | CHB | 22.4 | |  |  |  |

HC: healthy control; SR: spontaneous recovered control; CHB: chronic hepatitis B.
