# Supplementary material for: Association between Mannose-Binding Lectin Gene Polymorphisms and Hepatitis B Virus Infection: A Meta-Analysis
Source: PLoS One. 2013 Oct 8;8(10):e75371. doi: 10.1371/journal.pone.0075371 (PMC3792921; doi:10.1371/journal.pone.0075371)
Supplement: Table S3 — Distribution of polymorphisms of MBL2 promoter plus exon1 among HBV cases and controls in the meta-analysis. (DOC) [file pone.0075371.s003.doc]

Table S3 Distribution of polymorphisms of MBL2 promoter plus exon1 among HBV cases and controls in the meta-analysis.

| **Study** | **Samples** | **Total Number** | **O/O and XA/O ^a^** | **other genotypes^a^** |
| --- | --- | --- | --- | --- |
| **Thio CL 2005^[15]^** | SR | 301 | 39 | 262 |
|  | CHB | 179 | 34 | 145 |
| **Chong WP 2005^[16]^** | SR | 87 | 6 | 81 |
|  | CHB | 320 | 25 | 295 |
| **Fletcher GJ 2010^[20]^** | SR | 147 | 68 | 79 |
|  | CHB | 133 | 52 | 81 |
| **Chatzidaki V 2012^[22]^** | SR | 36 | 5 | 31 |
|  | CHB | 33 | 3 | 30 |

^a^ “O/O and XA/O” was considered as low MBL-producing genotypes in this analysis, while “other genotypes” was considered to be high MBL-producing. SR: spontaneous recovered control; CHB: chronic hepatitis B.
