# Supplementary material for: Association between Mannose-Binding Lectin Gene Polymorphisms and Hepatitis B Virus Infection: A Meta-Analysis
Source: PLoS One. 2013 Oct 8;8(10):e75371. doi: 10.1371/journal.pone.0075371 (PMC3792921; doi:10.1371/journal.pone.0075371)
Supplement: Table S4 — Non-superiority tests of mbl2 polymorphism in CHB as compared with HC. (DOC) [file pone.0075371.s004.doc]

Table S4 Non-superiority tests of mbl2 polymorphism in CHB as compared with HC.

| **Study** | **No. of mbl2 O allele carriers** | | **Delta of O-allele carrier frequency (%)** | **Nonsuperiority P-value H0: O**  **frequency, cases>controls +5%** |
| --- | --- | --- | --- | --- |
|  | **CHB (%)** | **HC (%)** |  |  |
| **Thomas HC 1996^[8]^** | 9(22.5) | 29(12.4) | 10.1% | 0.8061 |
| **Bellamy R 1998^[9]^** | 116(32.2) | 395(30.2) | 2.0% | 0.1354 |
| **Höhler T 1998^[10]^** | 31(25.4) | 31(24.2) | 1.2% | 0.2429 |
| **Shi H 2001^[12]^** | 99(17.4) | 61(20.3) | -2.9% | 0.2307 |
| **Song le H 2003^[1]^** | 3(6.0) | 9(4.0) | 2.0% | 0.1729 |
| **Filho RM 2010^[19]^** | 50(24.5) | 92(19.8) | 4.7% | 0.4632 |
| **Chatzidaki V 2012^[22]^** | 8(12.1) | 8(12.5) | -0.4% | 0.2113 |
| **Zheng RD 2012^[23]^** | 70(20.1) | 26(14.7) | 5.4% | 0.5462 |
| **Combined** | 1760(21.9) | 2888(22.4) | -0.5% | 0.0005 |

HC: healthy control; CHB: chronic hepatitis B.
