# Supplementary material for: Association between Mannose-Binding Lectin Gene Polymorphisms and Hepatitis B Virus Infection: A Meta-Analysis
Source: PLoS One. 2013 Oct 8;8(10):e75371. doi: 10.1371/journal.pone.0075371 (PMC3792921; doi:10.1371/journal.pone.0075371)
Supplement: Table S5 — Non-superiority tests of mbl2 polymorphism in CHB as compared with SR. (DOC) [file pone.0075371.s005.doc]

**Table S5 Non-superiority tests of mbl2 polymorphism in CHB as compared with SR.**

| **Study** | **No. of mbl2 O allele carriers** | | **Delta of O-allele carrier frequency (%)** | **Nonsuperiority P-value H0: O**  **frequency, cases>controls +5%** |
| --- | --- | --- | --- | --- |
|  | **CHB (%)** | **SR (%)** |  |  |
| **Thomas HC 1996^[8]^** | 16(24.2) | 8(21.1) | 3.1 | 0.4164 |
| **Bellamy R 1998^[9]^** | 116(32.2) | 93(29.6) | 2.6 | 0.2512 |
| **Höhler T 1998^[10]^** | 31(25.4) | 12(21.4) | 4.0 | 0.4414 |
| **Song le H 2003^[1]^** | 3(6) | 8(12.9) | -6.9 | 0.6317 |
| **Cheong JY 2005^[14]^** | 143(19.2) | 60(23.8) | -4.6 | 0.4443 |
| **Chen DQ 2010^[21]^** | 89(14.6) | 103(14.3) | 0.3 | 0.0084 |
| **Chatzidaki V 2012^[22]^** | 8(12.1) | 8(11.1) | 1.0 | 0.2323 |
| **Combined** | 406(20.1) | 292(19.3) | 0.9 | 0.0012 |

SR: spontaneous recovered control; CHB: chronic hepatitis B.
